# Distribution and role of peripheral arterial chemoreceptors in cardio-respiratory control of the South American rattlesnake (*Crotalus durissus*)

**DOI:** 10.1242/jeb.249222

**Published:** 2025-02-20

**Authors:** Catalina Reyes, Angelina Y. Fong, Cleo A. C. Leite, Augusto S. Abe, William K. Milsom

**Affiliations:** ^1^ School of Biological Sciences, University of California, San Diego, La Jolla, CA 92093, USA; ^2^ Department of Anatomy and Physiology, University of Melbourne, Parkville, VIC 3010, Australia; ^3^ Department of Physiological Sciences, Federal University of São Carlos (UFSCar), São Carlos, SP 13565-905, Brazil; ^4^ Departamento de Zoologia, Universidade Estadual Paulista, Rio Claro, São Paulo 13506-900, Brazil; ^5^ Department of Zoology, University of British Columbia, Vancouver, BC, Canada, V6T 1Z4

**Keywords:** Chemoreceptors, Carotid body, Pulmonary chemoreceptors, Aortic chemoreceptors, Cardiac shunt, Ventilation, Rattlesnake

## Abstract

Peripheral arterial chemoreceptors monitor the levels of arterial blood gases and adjust ventilation and perfusion to meet metabolic demands. These chemoreceptors are present in all vertebrates studied to date but have not been described fully in reptiles other than turtles. The goals of this study were to (1) identify functional chemosensory areas in the South American rattlesnake (*Crotalus durissus*), (2) determine the neurochemical content of putative chemosensory cells in these areas and (3) determine the role each area plays in ventilatory and cardiovascular control. To this end, rattlesnakes were instrumented with transonic flow probes, arterial catheters and subcutaneous impedance electrodes to measure shunt fraction, heart rate, blood pressure and ventilation. The catheters were placed at three putative chemosensory sites, the bases of the aortic arch and pulmonary artery, and the carotid bifurcation, for site-specific activation with sodium cyanide (NaCN). These same sites were subsequently examined using immunohistochemical markers for acetylcholine, tyrosine hydroxylase (the rate-limiting enzyme in catecholamine synthesis) and serotonin to identify putative oxygen-sensing cells. All three sites were chemosensory and stimulating each led to cardiovascular (shunt fraction and heart rate) and respiratory adjustments although not in an identical fashion. All three chemosensory areas contained cells positive for serotonin; however, cells positive for vesicular acetylcholine transporter (VAChT) were found only in the aorta and pulmonary artery. We found no labelling for tyrosine hydroxylase at any site.

## Teaser

**Highlighted Article:** Novel insights into reptilian chemoreceptors, identifying and characterizing three functional chemosensory areas in rattlesnakes and expanding our understanding of vertebrate respiratory and cardiovascular control mechanisms.

## INTRODUCTION

A primary role of the cardio-respiratory system is to supply oxygen to satisfy the metabolic demands of tissues. To fulfil this role, peripheral arterial chemoreceptors continuously monitor the levels of oxygen in the blood and adjust ventilation and perfusion to match oxygen supply to demand (Shelton et al., 1986; Gonzalez et al., 1994; Milsom, 1998; Milsom et al., 2022). Peripheral chemoreceptors have been found in all vertebrates studied so far (Adams, 1958; Ishii et al., 1966, 1985a,b; Ishii and Ishii, 1986; Rogers, 1967; Abdel-Magied and King, 1978; Lahiri et al., 1981; de Graaf, 1990; Gonzalez et al., 1994; Jonz and Nurse, 2003; Milsom et al., 2022). They are always associated with derivatives of pharyngeal arches and innervated by the glossopharyngeal (IX) and/or vagus (X) nerves (Milsom and Burleson, 2007). In reptiles, however, functional peripheral chemoreceptors have only been fully identified in turtles, located in the common carotid artery, aortic arch and pulmonary artery (derivatives of the 3rd, 4th and 6th pharyngeal arches, respectively) (Ishii et al., 1985b; Ishii and Ishii, 1986; Reyes et al., 2015). In lizards, anatomical evidence suggests the presence of chemoreceptor cells in the truncal region (Berger et al., 1982) and at the carotid bifurcation (Adams, 1962; Rogers, 1967; Reichert et al., 2015). Other areas have not yet been investigated in lizards. Nothing is known regarding peripheral arterial chemoreceptors in snakes and crocodilians.

Reptiles other than crocodilians have an undivided ventricle (Burggren, 1987; Hicks, 1998; Farrell et al., 1998), which allows them to shunt blood away from the lungs (R–L shunt), reducing arterial oxygen, or towards the lungs (L–R shunt), increasing arterial oxygen. Thus, in reptiles, adjustments in both ventilation and cardiac shunt regulate arterial oxygen content (Hicks and Wood, 1989; Wang and Hicks, 1996; Wang et al., 1997; Wood, 1982, 1984). The relative importance of the respiratory or cardiovascular systems to the regulation of blood gases depends largely on environmental and physiological conditions (normoxia, hypoxia, hypoxaemia and anaemia). For instance, a reduction in the R–L shunt increases oxygen delivery to the tissues in anaemic and normoxic turtles more effectively than an increase in ventilation (Wang and Hicks, 1996; Wang et al., 1997), as haemoglobin in blood leaving the lungs is already fully saturated (Wood, 1984). During hypoxia, when pulmonary venous blood is not fully saturated, arterial blood gases are more effectively regulated by increasing ventilation (Wang and Hicks, 1996). Similarly, anaemic toads increase heart rate, while hypoxia elicits changes in ventilation (Wang et al., 1994; Branco and Glass, 1995). Given these findings, Wang and collaborators (1994, 1997, 2004) proposed that amphibians and reptiles have anatomically distinct groups of chemoreceptors that sense arterial oxygen tension or oxygen content and adjust the respiratory and cardiovascular systems, respectively. To date, this hypothesis that the respiratory and cardiovascular systems are independently regulated by different groups of peripheral chemoreceptors has not been tested.

Very little is also known about the neurochemical content of putative oxygen-sensing cells in reptiles. There have been observations of nests of epithelioid cells in the carotid arch of lizards (Rogers, 1967; Berger et al., 1982) and granulated cell clusters in all the chemosensory areas of the tortoise (Kusakabe et al., 1988). Formaldehyde vapor exposure has revealed an incidence of cells containing biogenic amines (serotonin or catecholamines) in the carotid arch of lizards (Kobayashi, 1971) and the truncus and aorta of chelonians (Chiba and Yamauchi, 1973; Ishii et al., 1985b). In tegu lizards, cells at the first carotid bifurcation were immune-positive for vesicular acetylcholine transporter (VAChT) and serotonin (5-HT) but not tyrosine hydroxylase (TH) (Reichert et al., 2015). Furthermore, two populations of putative chemoreceptor cells were identified in turtles containing either VAChT or serotonin (Reyes et al., 2015).

Given the foregoing, the objectives of the present study were to (1) locate functional chemosensory areas in rattlesnakes, (2) characterize the cardio-respiratory reflex responses of each area to a hypoxaemia mimetic (sodium cyanide, NaCN) and (3) identify putative oxygen-sensing cells based on their neurochemical content and anatomical features. We hypothesized that, as in frogs (Reyes et al., 2014) and turtles (Reyes et al., 2015), rattlesnakes would have three distinct chemosensory areas located at the carotid bifurcation, and the bases of the aortic arch and pulmonary artery, and that each would possess glomus-like cells with similar neurotransmitter profiles to the chemosensory areas described in turtles (Reyes et al., 2015). Furthermore, we hypothesized that aortic and pulmonary chemoreceptors would primarily regulate the degree of intra-cardiac shunt, while carotid chemoreceptors would primarily regulate ventilation. To test these hypotheses, we located potential chemoreceptor areas through their innervation and association with derivatives of pharyngeal arches and quantified the cardio-respiratory reflex responses to NaCN stimulation at each site before and after selectively denervating each area. To identify putative chemoreceptor cells, we used markers for three neurotransmitters: acetylcholine, TH (the rate-limiting enzyme in catecholamine synthesis; Nurse, 2005, 2010) and serotonin. By establishing the location, neurochemical profiles and regulatory roles of peripheral chemoreceptors in snakes, we hope to broaden our understanding of phylogenetic patterns of chemosensing seen in vertebrates. We aimed to investigate whether O_2_-sensing structures are similar across vertebrates, and whether the location and reflex roles of distinct chemoreceptor groups have changed to control ventilatory and cardiovascular functions more effectively in animals with central vascular shunts.

**List of abbreviations**

5-HT: serotonin
*f*_H_: heart rate
*f*_R_: breathing frequency
*f*_R_^′^: instantaneous breathing frequency
MAP: mean arterial blood pressure
*P*_pul_: pulmonary pulse pressure
*P*_sys_: systemic pulse pressure
*Q̇*_Lao_: left aorta blood flow
*Q̇*_PA_: pulmonary artery blood flow
*Q̇*_pul_: pulmonary blood flow
*Q̇*_pul_/*Q̇*_sys_: shunt pattern
*Q̇*_sys_: systemic blood flow
*Q̇*_tot_: total cardiac output
*R*_pul_: pulmonary resistance
*R*_sys_: systemic resistance
TH: tyrosine hydroxylase
*V*_amp_: breathing amplitude
*V*_S,tot_: total stroke volume
*V̇*_tot_: total ventilation
VAChT: vesicular acetylcholine transporter

## MATERIALS AND METHODS

### Animals and holding conditions

South American rattlesnakes (*Crotalus durissus* Linnaeus 1758) (mean±s.e.m. mass 1.4±0.08 kg, *N*=22) were obtained from the Butantan Institute in São Paulo and transported to Universidade Estadual Paulista (UNESP, Rio Claro, SP, Brazil). The snakes were housed in a vivarium at room temperature (∼28°C) under a natural photoperiod. The holding and experimental procedures followed Canadian Council on Animal Care guidelines and were approved by the University of British Columbia Animal Care Committee – CA (animal care certificate no. A09-0233) and the Committee on Ethics in Animal Experimentation, UNESP – BR (03/08 CEEA).

### Surgery and instrumentation

Snakes were initially sedated by inhalation of CO_2_ until all righting reflexes disappeared. The animals were intubated and artificially ventilated using a mechanical ventilator (Harvard Apparatus, Holliston, MA, USA) set at a frequency of 6 breaths min^−1^ and a tidal volume of 25 ml kg^−1^ (Galli et al., 2005, 2007). During surgery, anaesthesia was maintained with isoflurane (0.5–1%; Baxter Healthcare Corporation, Deerfield, IL, USA). A 7–8 cm ventral incision was made cranial to the heart and two 1.5–2R and 2.5R ultrasound, transit-time flow probes (Transonic System, Inc., Ithaca, NY, USA) were placed around the pulmonary artery and left aorta to measure pulmonary (*Q̇*_pul_) and systemic (*Q̇*_sys_) blood flow, respectively. Acoustical gel was infused around the flow probes to enhance the signal. The flow probes were connected to a dual-channel blood flow meter (Transonic T206). Arterial catheters filled with heparinized saline were occlusively placed in a side branch of the pulmonary and caudal arteries for measurement of pulmonary (*P*_pul_) and systemic (*P*_sys_) pulse pressure, respectively. Both catheters were connected to pressure transducers and the signals were amplified with a Gould DC amplifier. Both pressure transducers (systemic blood pressure: Narco Biosystems, Narco Scientific, Houston, TX, USA, model 320-1000E; pulmonary blood pressure: Statham, Hato Rey, Puerto Rico, model P23Db) were placed at the level of the heart and were calibrated daily using an aneroid barometer. Heart rate was derived from the blood pressure traces and ventilation was inferred from impedance measured with a set of needle electrodes placed subcutaneously.

Snakes were divided into two experimental groups. In group 1 (*N*=10 aorta and *N*=12 pulmonary artery), for site-specific stimulation of putative aortic and pulmonary chemoreceptors, PE50 and PE10, polyethylene catheters filled with heparinized saline were advanced into the right aortic arch through the vertebral artery and into the pulmonary artery at the base of the heart through a small branch of the pulmonary artery, respectively. In group 2 (*N*=10), the left carotid artery was non-occlusively cannulated (percutaneous cannula) and the catheter was advanced towards the carotid bifurcation, for specific stimulation of putative carotid chemoreceptors. The catheters and leads of each probe were secured to the skin with suture, and local analgesia (xylocaine) was applied to the incision. Animals were placed in dark plastic boxes and allowed to recover overnight. All experiments were performed at room temperature on fully recovered snakes. At the end of the experiment, animals were euthanized and the position of the catheters was verified. The left carotid bifurcation, pulmonary artery, right and left aortas, jugular ganglia and the lungs of five animals were collected for immunohistochemical analysis.

### Denervation of carotid or pulmonary and aortic branches of the vagus

Denervation studies were performed to confirm that the responses observed after focal stimulation with NaCN originated from peripheral chemoreceptor activation. The denervation procedures were performed on a separate group of snakes from those used in the intact experiments. In group 1, branches of the vagus innervating the base of the pulmonary artery and aortic arch were severed (*N*=3), while the vagus trunci and the branch that innervates the proximal portion of the pulmonary artery were left intact. It was impossible to discriminate between the small branches of the vagus that innervate the pulmonary artery and aorta, which run together. In group 2, branches of the vagus innervating the carotid bifurcation and nearby areas were severed (*N*=4) and the main trunk of the vagus was left intact, while a heavily vascularized and innervated epithelial body sitting at the carotid bifurcation was excised. We confirmed that denervating a particular chemoreceptor site did not affect the shunting capability by stimulating other intact chemoreceptor groups and observing the shunting reflex.

### NaCN injection protocol

NaCN injections were used to determine the presence of chemoreceptors in the putative chemosensory areas. The volume of NaCN infused in the cannula was small (0.1 ml) to prevent mechanoreceptor stimulation. A preliminary dose–response (0.1, 0.3 and 0.5 mg in 0.1 ml) experiment was conducted in three animals to determine the concentration of NaCN to be used in the experiments. The animals received bolus injections of NaCN (0.5 mg, volume injected 0.1 ml; 380970, Sigma-Aldrich) through the catheters in the vertebral and side branch of the pulmonary artery (group 1) and the carotid artery (group 2). Injections of NaCN were followed by 0.8 ml of saline to ensure that no NaCN remained in the cannula. This value was determined by measuring the volume of the catheters. Sham injections of saline (0.9 and 2 ml; NaCl, S7653, Sigma-Aldrich) were used to further confirm that the responses observed were not an artefact of the injection volume (Table S1). The order of the injections was randomized and at least 20 min elapsed between injections so that blood flow, heart rate and blood pressure returned to baseline.

### Data analysis and statistics

Mean blood pressure, blood flow, heart rate, ventilation amplitude and breathing frequency were recorded over a period of 10 min before the stimulus was applied and until all physiological variables returned to baseline using WINDAQ acquisition software (version 2.19, Dataq Instruments, Akron, OH, USA) sampling at a rate of 250 Hz per channel. As rattlesnakes only possess one pulmonary artery, measurements of blood flow in the pulmonary artery (*Q̇*_PA_) represent *Q̇*_pul_. *Q̇*_sys_ was estimated as 3.3 times left aorta blood flow (*Q̇*_LAo_) (Galli et al., 2005). Total cardiac output (*Q̇*_tot_) was calculated as *Q̇*_sys_+*Q̇*_pul_. Shunt pattern was calculated as *Q̇*_pul_/*Q̇*_sys_. Total stroke volume (*V*_S,tot_; pulmonary+systemic) was calculated as *Q̇*_tot_/*f*_H_ (where *f*_H_ is heart rate calculated from the blood pressure trace). Systemic resistance (*R*_sys_) and pulmonary resistance (*R*_pul_) were calculated as *P*_sys_/*Q̇*_sys_ and *P*_pul_/*Q̇*_pul_, respectively, assuming that central venous blood pressures were negligible. Total ventilation [*V̇*_tot_; amplitude (*V*_amp_)×breathing frequency (*f*_R_)] and amplitude are expressed as percentage values relative to the pre-injection values (control). We used an alpha critical level of 0.05 to determine statistical significance.

#### Effects over time of NaCN and saline injections

All cardiovascular variables were averaged over the 2 min before NaCN or saline injections and over 1 min intervals after the injection. Respiratory data were averaged over the 4 min before stimulus application and over 2 min intervals after the injection.

All values are presented as means±s.e.m. We tested for differences between control values (before injection) and values after NaCN or saline injections on blood flow, blood pressure, heart rate and breathing frequency with a one-way repeated measures ANOVA. Holm–Šidák multiple comparisons tests were used to determine pairwise differences. Data that did not meet the assumptions of normal distribution or equal variances were natural log (ln) transformed or tested for significant differences with a non-parametric Friedman repeated measures analysis of variance. We tested whether the mean of the post injected values (relative values) for breathing amplitude and total ventilation were higher than 100% (control) using a one-sample *t*-test.

#### Peak response to NaCN injections

Respiratory data were also analysed to show the peak response after NaCN injections as the time to peak response to the bolus injections varied between animals. The purpose of this analysis was to ensure that rapid responses were not lost by averaging the respiratory data over 2 min intervals. The instantaneous breathing frequency (*f*_R_′) was calculated as 60 s min^−1^/inter-breath interval (s breath^−1^). The *f*_R_′ and breath amplitude were averaged over the 4 min prior to the NaCN bolus injection. The peak response to the NaCN stimulus was obtained using the breath with the maximum instantaneous breathing frequency and its corresponding amplitude. Values are presented as means±s.e.m. We tested for differences between control values (prior to NaCN injection) and the peak response (maximum *f*_R_′) using a paired *t*-test. Effects of NaCN on the corresponding breathing amplitude and total ventilation were shown as a relative change from control (4 min average before injection) and differences were tested using a one-sample Wilcoxon signed rank test. Sigma Stat (version 3.11, Systat Software, Evanston, IL, USA) or R (http://www.R-project.org/) was used for all statistical analyses.

### Immunohistochemical and histological analysis

#### Tissue preparation

The right and left carotid bifurcation and segments of the left common carotid artery, pulmonary artery and right and left aorta (*N*=5) were removed and flushed with heparinized saline (100 UI ml^−1^) using a blunt 21-gauge needle connected to a syringe until the blood vessels appeared clear of blood. The jugular ganglia and lungs of two animals were also collected to use as positive immunohistochemical controls (see description of controls in ‘Immunohistochemistry’, below). The tissue was pinned, immersed in paraformaldehyde (PFA; 4%) in 0.1 mol l^−1^ phosphate-buffered saline (PBS; Na_2_HPO_4_, 13.4 g l^−1^; NaH_2_PO, 6 g l^−1^; NaCl, 9 g l^−1^; buffered to pH of 7.4 with NaOH) and stored at 4°C until it was shipped to the University of California, San Diego, CA, USA. Upon arrival, the tissue was washed in PBS and cryoprotected in 30% sucrose buffer and frozen in Tissue Tek^®^ (Sakura, San Marcos, CA, USA) at −80°C. Longitudinal sections (18 μm thick) were made using a cryostat (CM3050, Leica Microsystems, Nussloch, Germany) and serially mounted on Superfrost^®^ plus slides (VWR International, West Chester, PA, USA). Slide-mounted sections were immediately processed for immunohistochemistry or stored in a −80°C freezer until needed.

#### Haematoxylin and eosin histology

Tissue sections from the pulmonary artery, aortas and right carotid bifurcation were stained with haematoxylin and eosin (H&E) to visualize the internal structure of blood vessels and identify the location of putative oxygen-sensing cells. Fixed tissue samples were dehydrated through a graded ethanol series (70%, 80%, 95%, 100%; A407, Fisher Scientific, Ottawa, ON, Canada), cleared in xylene (Fisher Scientific) and embedded in paraffin at 56°C. Longitudinal sections (20 µm thick) were cut through representative regions of the central vasculature using a Leica rotary microtome (RM2255, Leica Microsystems, Wetzlar, Germany) and mounted onto slides. The sections were processed by UC San Diego Moores Cancer Center (San Diego, CA, USA) using standard H&E staining protocols, followed by dehydration and coverslipping (#1, Fisher Scientific) with Permount (SP15-500, Fisher Scientific).

#### Immunohistochemistry

Slide-mounted tissue was washed in PBS; the buffer was pipetted in and out of the glass holders to prevent the delicate internal structure of the arteries from breaking off from the slides. Following the washes, the sectioned tissue was blocked in 10% normal donkey serum (NDS; Jackson Laboratories, distributed by Cedarlane Laboratories, Hornby, ON, Canada) for 1 h. Primary antibodies (mouse anti-TH and goat anti-5-HT, ImmunoStar; rabbit anti-VAChT, Novus Biologicals) were diluted (PBS, 0.3% Triton X-100, 2% NDS) according to optimal concentrations determined by previous dilution curves (Table 1). Slides were incubated with the primary antibody (individually or in combination) for 48 h at room temperature and then washed in PBS. Following the washes, slides were incubated in the dark with fluorescently labelled secondary antibodies diluted in PBS (with 0.3% Triton-X and 2% NDS) (Table 1) for 2 h and subsequently washed in PBS. DAPI was used to visualize cell nuclei (Vectashield with DAPI, Vector Laboratories, Burlington, ON, Canada). Coverslips (#1.5, Fisher Scientific) were mounted with Vectashield (Vector Laboratories) to reduce photobleaching and the coverslips were sealed with nail polish. Processed slide-mounted tissue was stored at 4°C in the dark prior to imaging.

**Table 1.** **Primary and** **secondary antibodies used for immunohistochemistry**

Antibody	Host species and antigen	Immunogen	Fluorophore	Dilution	Manufacturer, cat. no.	RRID
Primary	5-HT, goat (polyclonal)^c^	5-HT coupled to BSA with PFA		1:650	ImmunoStar, 20079	AB_572262
	TH, mouse (monoclonal; IgG1)^b^	Full-length TH purified from rat PC12 cells		1:50	ImmunoStar, 22941	AB_572268
	VAChT, rabbit (polyclonal)^a^	Synthetic peptide corresponding to the C-terminus of mouse VAChT conjugated to immunogenic carrier protein		1:300	Novus Biologicals, NBP1-46776	AB_11003138
Secondary	Donkey anti-rabbit IgG (H+L)^a^		Alexa Fluor^®^ 488	1:200	Invitrogen, A-21206	AB_2535792
	Donkey anti-mouse IgG (H+L)^b^		Alexa Fluor^®^ 488	1:200	Invitrogen, A-21202	AB_141607
	Donkey anti-goat IgG (H+L)^c^		Alexa Fluor^®^ 555	1:200	Invitrogen, A-21432	AB_141788

^a,b,c^Secondary antisera antigen corresponds with primary antibody host. Secondary antisera were conjugated with a fluorescent marker. 5-HT, serotonin; TH, tyrosine hydroxylase; VAChT, vesicular acetylcholine transporter.

Controls consisted of excluding the primary antibody to control for non-specific binding of the secondary antibody. To control for interactions between antibodies, single-labelled slides were processed in each run. Positive controls for VAChT and 5-HT primary antibodies were performed using the jugular ganglion and lung, respectively.

#### Microscopy for cryo-sectioned tissue

Sectioned tissue was observed using an epifluorescence light microscope (Zeiss AxioObserver Z1, Carl Zeiss) connected to a halide lamp and equipped with 488049-9901 FL Filter Set 49, 489038-9901 FL Filter Set 38 HE and 489043-9901 FL Filter Set 43 HE filters to detect DAPI, GFP and Cy3, respectively. Images were captured using AxioVision software (Rel. 4.8.3, Carl Zeiss Microscopy). Representative sections were further examined using a spinning disk microscope (Perkin Elmer Ultraview VOX Spinning Disk Confocal, Waltham, MA, USA), equipped with 405, 488 and 561 nm lasers and 527/55, 445/60 or 615/70, 525/50 or 640/120 filters for detection of GFP, DAPI and rhodamine. Z-stacks of 117–179 optical sections 0.2 µm apart were captured using Leica multi-immersion ×20 and ×63 glycerol objectives and a Hamamatsu C9100-50 camera.

#### Quantification of cell size

We measured the diameters of different cell types to further characterize candidate oxygen-sensing cells. Only cells where we could identify the nuclei and clearly see the entire labelled cytoplasm were used for quantification. Ten 5-HT and 10 VAChT-immunoreactive (IR) cells were measured in different sections of the carotid bifurcation, right aorta and pulmonary artery of four animals using ImageJ software. All data are presented as mean±s.e.m. cell size (µm).

## RESULTS

### Anatomy and innervation of the central vasculature

**Fig. 1.**
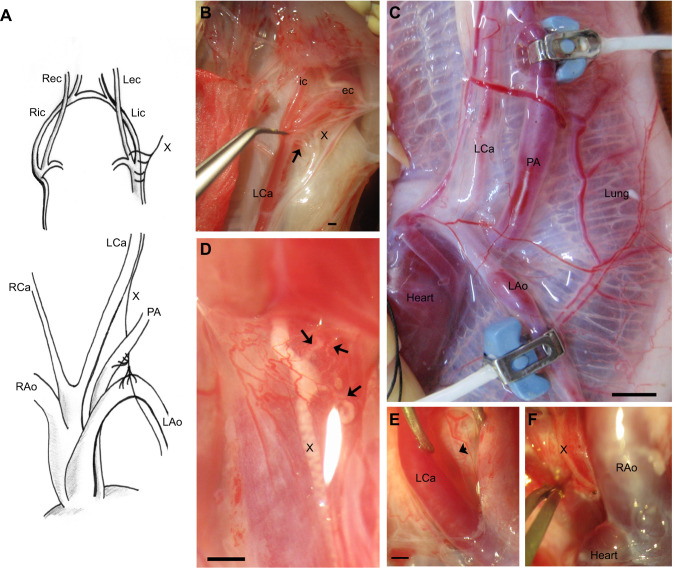
**Anatomy** **and innervation of the central vasculature of *Crotalus durissus*.** Schematic diagram (A) and pictures (B–F) showing the anatomy (C) and innervation by the vagus nerve (arrows) of the carotid bifurcation (B,D) of *C. durissus*. The vagus nerve runs caudally (E) by the carotid artery towards the aorta, pulmonary artery and heart (F). ic, internal carotid artery; ec, external carotid artery; Ca, carotid artery; Ao, aorta; PA, pulmonary artery; X, vagus nerve; L, left side of the animal; R, right side of the animal. Scale bars: 1 mm (B, D–F) and 5 mm (C).

In rattlesnakes, the left and right aortas and single pulmonary artery originate from the ventricle (Fig. 1A,C). The common carotid arteries arise from the right aorta. The right carotid artery is truncated, while the left extends to the head, bifurcating into the internal and external carotid arteries (Fig. 1A,B). An anastomosis connects the left carotid artery to the right carotid artery’s rostral extension, supplying the right internal and external carotid arteries.

To identify potential chemosensory areas in the snakes, we traced the innervation of the glossopharyngeal (IX)/vagus (X) nerves to the carotid bifurcation, aorta and pulmonary artery. In rattlesnakes, the vagus and glossopharyngeal nerves join after leaving the skull. Small vagal branches innervate the carotid bifurcation (Fig. 1A,B,D) and a nearby vascularized glandular body (not shown). The left vagus nerve continues caudally alongside the left carotid artery (Fig. 1E, arrowhead), while the right vagus nerve supplies the remnant of the right carotid. Three tracheal branches arise from the vagus trunk to innervate the trachea. Numerous small branches arise from the vagus nerve to innervate the aorta and pulmonary artery in the region proximal to the heart (Fig. 1A,F). Thus, each of the carotid bifurcation, aorta and pulmonary artery are extensively innervated by the vagus/glossopharyngeal nerve.

### Chemosensory areas in rattlesnakes

The nerve tracing indicated that the left carotid bifurcation, aortic arch and pulmonary artery could all be functional chemosensory areas in rattlesnakes. To test this, we recorded cardio-respiratory variables while injecting each site individually with NaCN, a hypoxaemia mimetic. We determined that the cardio-respiratory adjustments produced by chemoreceptor stimulation were not due to an injection effect, as saline injections of 0.9 ml (same volume as NaCN injections) or 2 ml did not trigger changes in any of the cardiovascular or respiratory variables (Table S1). We confirmed that the cardio-respiratory reflexes were produced by stimulation at specific chemoreceptor sites by the absence of a reflex response after NaCN stimulation in animals where the small vagal branches innervating either the carotid bifurcation or the aorta and pulmonary artery were severed (*N*=4, carotid; *N*=3, aorta/pulmonary artery). Furthermore, we verified that the ability to shunt was not affected by denervation of a particular chemoreceptor site, by stimulating intact chemoreceptor groups at other sites to elicit a reflex response.

#### Cardiovascular responses to chemoreceptor activation

##### Cardiovascular control by peripheral chemoreceptors in the carotid bifurcation

**Fig. 2.**
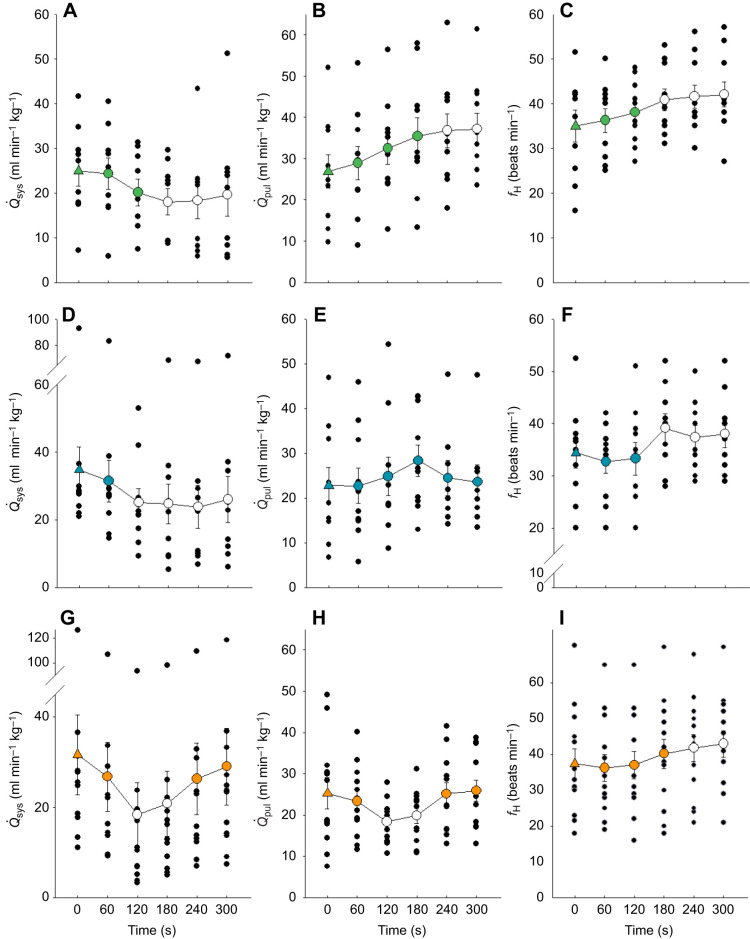
**Changes in systemic blood flow (*Q̇*_sys_; left), pulmonary blood flow (*Q̇*_pul_; centre) and heart rate (*f*_H_; right) following NaCN injection in the carotid bifurcation, aorta and pulmonary artery of *C. durissus*.** Filled triangles indicate pre-injection values (control) and circles indicate post-injection values averaged over 1 min (means±s.e.m.) for the carotid bifurcation (A–C), aorta (D–F) and pulmonary artery (G–I); individual data points are shown in black. Open circles denote values that are significantly different from the control (Holm–Šidák pairwise comparison) (*N*=10 carotid and aortic groups; *N*=12 pulmonary group). The *y*-axis in some panels has been truncated.

**Fig. 3.**
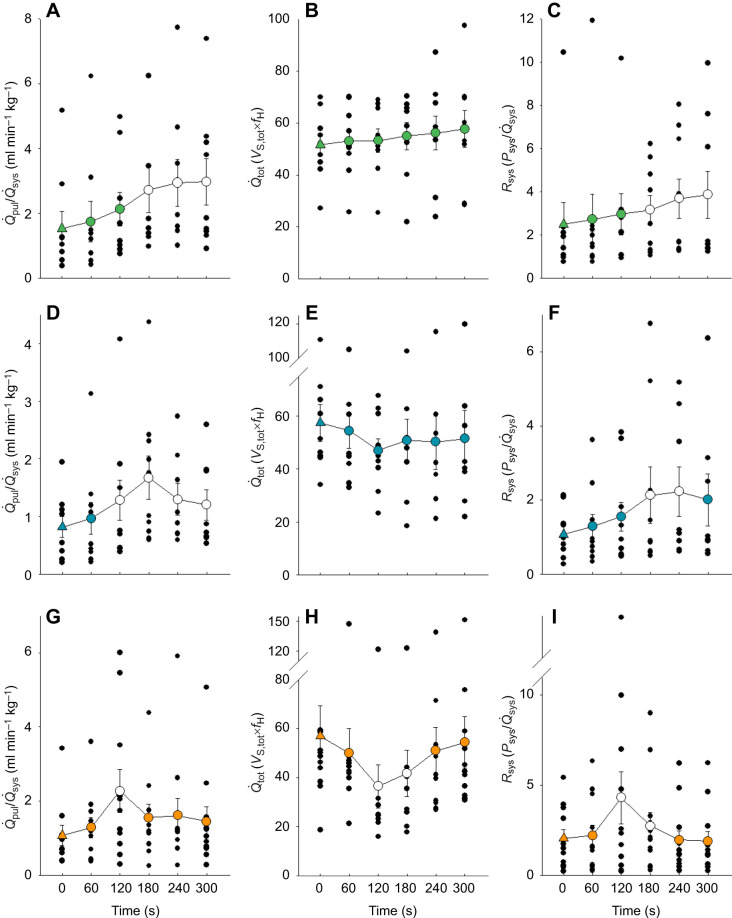
**Changes** **in shunt fraction (*Q̇*_pul_/*Q̇*_sys_;** **left), cardiac output (*Q̇*_tot_; centre) and systemic resistance (*R*_sys_; right) following NaCN injection in the carotid bifurcation, aorta and pulmonary artery of *C. durissus*.** Filled triangles indicate pre-injection values (control) and circles indicate post-injection values averaged over 1 min (means±s.e.m.) for the carotid bifurcation (A–C), aorta (D–F) and pulmonary artery (G–I); individual data points are shown in black. Open circles denote values that are significantly different from the control (Holm–Šidák pairwise comparison) (*N*=10 carotid and aortic groups; *N*=12 pulmonary group). The *y*-axis in some panels has been truncated. *V̇*_S,tot_, stroke volume; *P*_sys_, systemic pulse pressure.

**Fig. 4.**
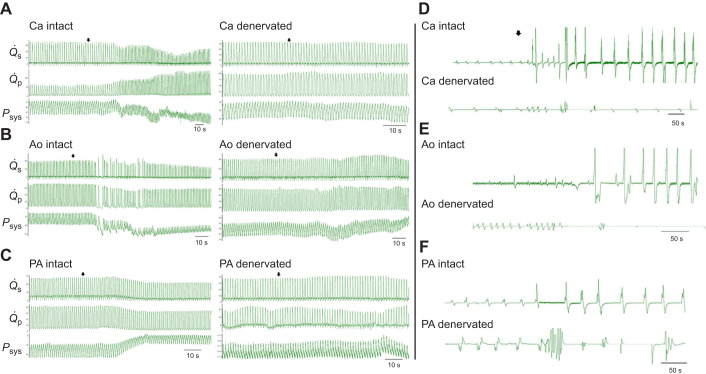
**Original** **traces showing the effects of NaCN injection (arrows) in the carotid bifurcation, aorta and pulmonary artery on haemodynamic variables (left) and ventilation and breathing pattern (right) of intact and denervated *C. durissus*.** Ca, carotid (A,D); Ao, aorta (B,E); PA, pulmonary artery (C,F).

NaCN injections into the carotid artery caused systemic blood flow (*Q̇*_sys_) to fall and pulmonary blood flow (*Q̇*_pul_) to rise (Fig. 2A,B), leading to a reduction in the right to left (R–L) shunt (increase in *Q̇*_pul_/*Q̇*_sys_) (Fig. 3A). Heart rate (*f*_H_) increased significantly (Fig. 2C). Stroke volume (*V*_S,tot_, Table 2) and cardiac output (*Q̇*_tot_, Fig. 3B) remained unchanged after carotid chemoreceptor stimulation. NaCN injections did not affect systemic mean arterial pressure (MAP) but caused a significant fall in systemic pulse pressure (*P*_sys_) (Table 2). Systemic resistance (*R*_sys_) was elevated compared with pre-injection values (Fig. 3C). After denervation of the carotid bifurcation, NaCN injection did not elicit any reflex response (Fig. 4A), confirming that site-specific carotid chemoreceptor activation caused the cardiovascular changes.

**Table 2.** **Effects** **of NaCN on cardiovascular variables**

Time	*V*_S,tot_ (ml kg^−1^)			MAP (mmHg)			*P*_sys_ (mmHg)		
	Ca	Ao	PA	Ca	Ao	PA	Ca	Ao	PA
Control	1.5±0.2	1.7±0.2	1.5±0.2	41.2±3.7	31.2±4	43.2±6.4	9.6±1.4	8.3±1.9	12.3±1.7
60 s	1.5±0.2	1.7±0.2	1.4±0.1	41.9±3.8	30.9±3.9	38.4±6.5	9.5±1.4	7.7±1.6	13.2±2.4*
120 s	1.4±0.2	1.5±0.2	1.0±0.2*	41.9±3.9	29.1±3.6	31.5±4.7*	7.5±1.3*	6.4±1.3*	9.8±2.4*
180 s	1.4±0.2	1.4±0.2*	1.1±0.2*	43.2±4.2	31.2±3.9	30.0±3.7*	7.2±1.2*	6.5±1.5*	9.9±2.5*
240 s	1.4±0.2	1.4±0.3*	1.3±0.2	44.2±4.1	33.1±3.5	31.0±3.7*	7.6±1.4*	6.1±1.7*	10.7±2.2*
300 s	1.5±0.3	1.4±0.2*	1.3±0.2	44.4±3.8	32.5±3.4	32.8±4.1*	8±1.5	6.2±1.7*	11.3±2.2*

*V*_S,tot_, stroke volume; MAP, mean arterial pressure; *P*_sys_, systemic pulse pressure; Ca, carotid artery; Ao, aorta; PA, pulmonary artery. Values are means±s.e.m. (*N*=10 carotid and aortic groups; *N*=12 pulmonary group). *Significant difference from pre-injection value (control, Holm–Šidák pairwise comparison).

##### Cardiovascular control by aortic peripheral chemoreceptors

Activation of aortic chemoreceptors led to a significant decrease in *Q̇*_sys_, while *Q̇*_pul_ did not change (Fig. 2D,E). This resulted in a significant increase in *Q̇*_pul_/*Q̇*_sys_ and a reduction in the R–L shunt (Fig. 3D). Although the immediate response to NaCN was a slight fall in *f*_H_ and skipped heart beats in some of the snakes, a marked tachycardia and reduced stroke volume were observed after 3 min of stimulus application (Fig. 2F, Table 2). Cardiac output and MAP did not change (Fig. 3E, Table 2). NaCN caused a reduction in *P*_sys_ (Table 2) and an increase in systemic resistance (*R*_sys_) (Fig. 3F). After denervation of this area, all cardiovascular responses to NaCN were abolished (Fig. 4B).

##### Cardiovascular control by pulmonary peripheral chemoreceptors

NaCN stimulation of pulmonary chemoreceptors caused a significant fall in both *Q̇*_sys_ and *Q̇*_pul_ (Fig. 2G,H). As *Q̇*_sys_ was reduced proportionately more than *Q̇*_pul_, shunt fraction increased significantly, and the R–L shunt was reduced (Fig. 3G). The fall in systemic flow was presumably caused by the increase in *R*_sys_ (Fig. 3I). As in the other two areas, NaCN injection led to a significant increase in *f*_H_, although only after a slight fall in the first 2 min following NaCN stimulation (Fig. 2I). Stimulation of chemoreceptors in the pulmonary artery led to a decrease in *V*_S,tot_ (Table 2), *Q̇*_tot_ (Fig. 3H), systemic blood pressure and *P*_sys_ (Table 2). All cardiovascular responses to NaCN were abolished after denervation (Fig. 4C).

#### Effects of chemoreceptor stimulation on respiratory control

**Fig. 5.**
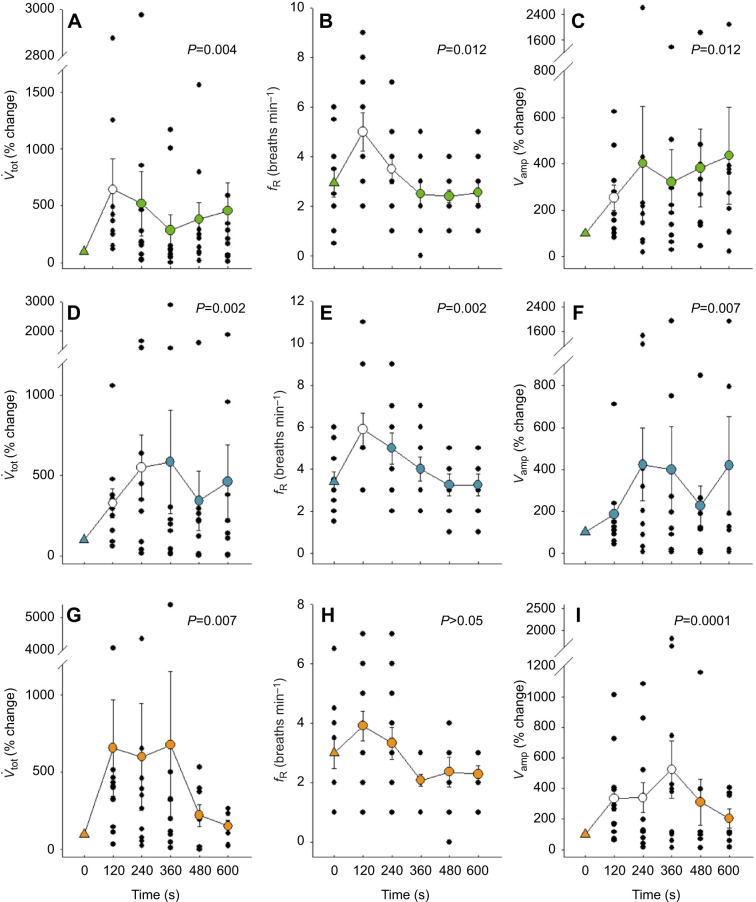
**Changes** **in total ventilation (*V̇*_tot_; left), breathing frequency (*f*_R_; centre) and breathing amplitude (*V*_amp_; right) following NaCN injection in the carotid bifurcation, aorta and pulmonary artery of *C. durissus*.** *V̇*_tot_ and *V*_amp_ are expressed as percentage values relative to the pre-injection values (control). Filled triangles indicate pre-injection values and circles indicate post-injection values averaged over 2 min (means±s.e.m.) for the carotid bifurcation (A–C), aorta (D–F) and pulmonary artery (G–I). Individual data points are shown in black. Open circles denote values that are significantly different from the control (*f*_R_, Holm–Šidák pairwise comparison; *V*_amp_ and *V̇*_tot_, one-sample *t*-test) (*N*=10 carotid and aortic groups; *N*=12 pulmonary group). The *y*-axis in some figures has been truncated.

NaCN injection into all three areas caused ventilation (*V̇*_tot_) to increase (Fig. 5A,D,G). In the carotid and aortic groups, changes in ventilation were due to significant increases in breathing frequency (*f*_R_) (Fig. 5B,E) and amplitude (*V*_amp_) (Fig. 5C,F). The increase in ventilation in response to NaCN injection in the pulmonary artery was caused mainly by a rise in *V*_amp_ (Fig. 5H,I). After selective denervation of the chemosensory sites, ventilation did not change. In fact, NaCN injection in the aorta of some denervated rattlesnakes inhibited ventilation, by a mechanism that remains unknown (Fig. 4D–F).

#### Effects of chemoreceptor stimulation on the respiratory peak response

NaCN injections triggered a significant increase in the peak ventilatory response in all three areas (carotid bifurcation *P*=0.002, aorta *P*=0.006, pulmonary artery *P*=0.002, one-sample Wilcoxon signed rank test). The change in ventilation resulted from a significant increase in the instantaneous breathing frequency (carotid bifurcation *P*=0.002, aorta *P*=0.005, pulmonary artery *P*=0.005, paired *t*-test). A significant increase in the breathing amplitude after NaCN stimulation was only observed in the pulmonary area (Table 3).

**Table 3.** Effects of NaCN on peak respiratory variables

Area	*V̇*_tot_ (% change)		*f*_R_′ (breaths min^−1^)		*V*_amp_ (% change)	
	Control	Peak response	Control	Peak response	Control	Peak response
Ca	100	2440.8±1361.9*	1.6±0.4	6.5±1.1*	100	408.9±177.9
Ao	100	1519.1±959.3*	2.1±0.3	7.3±1.6*	100	331.9±180.8
PA	100	5962.2±3914.3*	1.6±0.3	5.7±1.1*	100	537.6±218.3*

*V̇*_tot_, total ventilation; *f*_R_′, instantaneous breathing frequency; *V*_amp_, breathing amplitude; Ca, carotid artery; Ao, aorta; PA, pulmonary artery. *f*_R_′ control is the average pre-injection value and peak response is the maximum post-injection value. *V*_amp_ is the corresponding amplitude to the maximum instantaneous breathing frequency. *V̇*_tot_ and *V*_amp_ peak response is expressed as a percentage relative to the pre-injection (control) value. Values are means±s.e.m. (*N*=10 carotid and aortic groups; *N*=12 pulmonary group). *Significant difference from the control (*f*_R_′, paired *t*-test; *V̇*_tot_ and *V*_amp_, one-sample Wilcoxon signed rank test).

### Neurochemical content of the chemosensory areas

#### Controls for immunohistochemistry

Neuroepithelial bodies in the lung of rattlesnakes labelled for serotonin. VAChT-labelled neurons were observed in the jugular ganglion of the vagus/glossopharyngeal nerve (Fig. S1). Omission of the primary antibody did not result in detectable labelling in any of the immunohistochemical runs. Results from single and doubly labelled slides did not differ. These controls confirm that the procedures and antibodies used in this study are specific and effective in snakes.

#### Neurochemical content of the carotid bifurcation

**Fig. 6.**
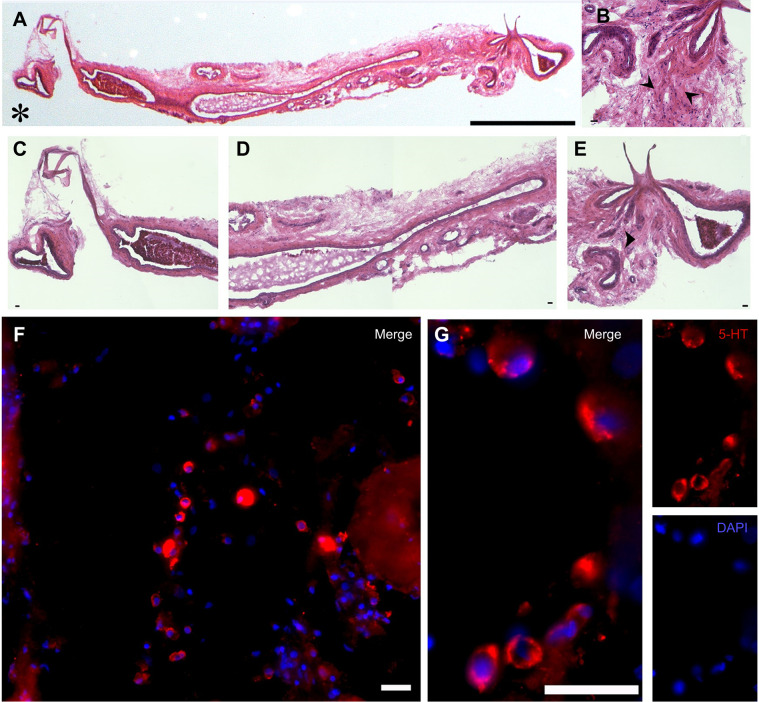
**Putative oxygen-sensing cells in a segment of the left carotid artery and the carotid bifurcation of *C. durissus*.** (A) Montage of a longitudinal section (20 µm thick) of the carotid bifurcation stained with haematoxylin and eosin (H&E). Asterisk indicates the heart end). (C–E) H&E-stained longitudinal sections showing regions of A at higher magnification. (B) Higher magnification of E showing the carotid bifurcation. Arrowheads indicate the location of 5-HT-immunoreactive (IR) cells. (F,G) Immunolabelling for 5-HT (red) and a nuclear stain (DAPI, blue). The images from the red and blue channels are shown separately and as a merged image. Scale bars: 1 mm (A), 25 µm (B–G).

The internal structure of the common carotid artery at the carotid bifurcation consists of spongy tissue surrounding a series of lumen sinusoids (Fig. 6A). We found numerous single 5-HT-IR cells embedded in the vessel wall at the carotid bifurcation (Fig. 6F, arrowheads in B and E) and a lower density of 5-HT-IR cells in the carotid artery just proximal to the bifurcation (Fig. 6D). 5-HT-IR cells were oval and 11.7±0.3 µm long (Fig. 6G). Serotonergic cells were not found in the carotid artery between the heart and the area just proximal to the bifurcation (data not shown). We saw a single positive VAChT cell in only one animal and in this one case, the two neurotransmitters (ACh and 5-HT) colocalized (data not shown).

#### Neurochemical content of the aorta

**Fig. 7.**
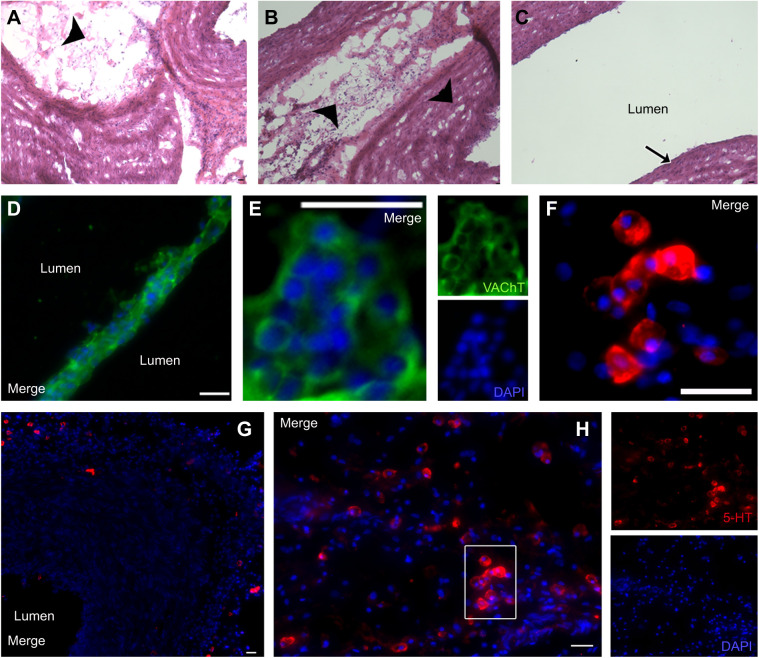
**Putative oxygen-sensing cells in the aorta of *C. durissus*.** (A–C) H&E-stained longitudinal sections (20 µm thick) of the aorta showing regions proximal to the heart (A, heart end). Arrowheads in A and B indicate the spongy tissue and vessel wall where 5-HT-IR cells are found. Arrow in C indicates the location of vesicular acetylcholine transporter (VAChT)-IR cells. (D,E) Immunolabelling for VAChT (green) and a nuclear stain (DAPI, blue). (E) The images from the red and green channels are shown separately and merged. (F–H) Immunolabelling for 5-HT (red) and a nuclear stain (DAPI, blue). (G) 5-HT-IR cells embedded in the vessel wall (right arrowhead in B). (H) 5-HT-IR cells in the spongy tissue indicated by the arrowhead in A and the left arrowhead in B; the outlined region is enlarged in F. The images from the red and blue channels are shown separately and as a merged image in H. Scale bars: 25 µm.

We found two cell populations in the aorta of rattlesnakes. One cell type labelled for VAChT and was characterized by clusters of oval cells (averaging 10.6±0.2 µm in diameter) with large nuclei (Fig. 7D,E). They were found in the spongy tissue inside the lumen of the blood vessel (Fig. 7C, arrow showing general location). A second cell type labelled for serotonin and was characterized by oval cells arranged singly or in groups of 2 or 3 (12±0.3 µm) (Fig. 7F). These cells were found embedded in the vessel wall throughout the aorta (Fig. 7B arrowheads, G), but their density was higher in the spongy tissue close to the heart (Fig. 7A,B arrowheads, H). 5-HT-IR cells and clusters of VAChT-IR cells were observed in proximity to each other within the same region. However, these two cell populations remained distinctly separate, with no instances of overlapping expression detected.

#### Neurochemical content of the pulmonary artery

**Fig. 8.**
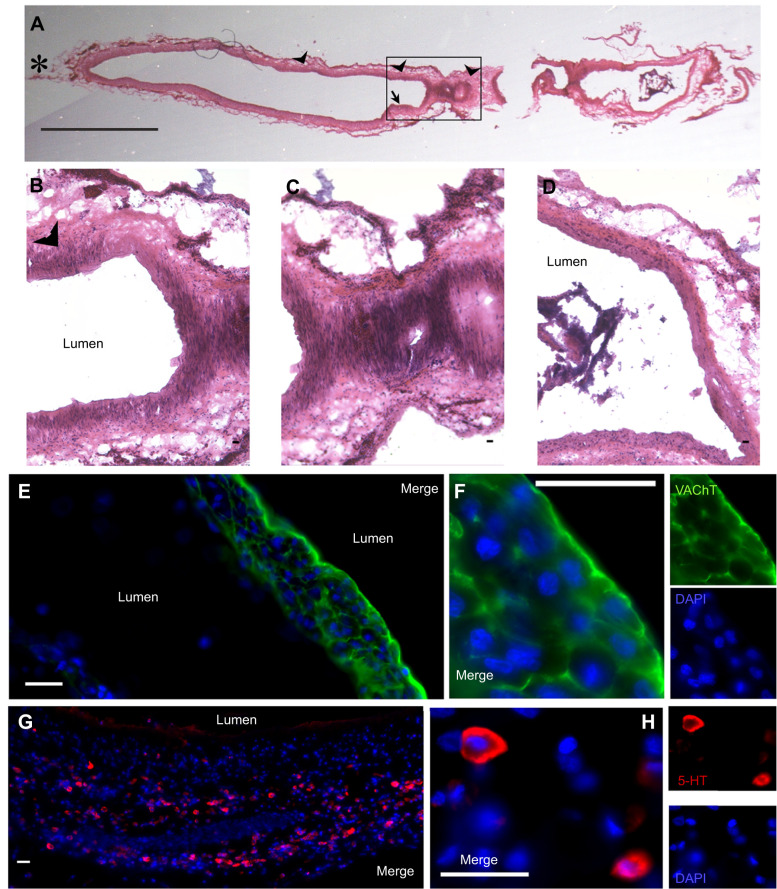
**Putative oxygen-sensing cells in the pulmonary artery of *C. durissus*.** (A) Montage of a longitudinal section (20 µm thick) of the pulmonary artery stained with H&E (asterisk indicates heart end). Arrowheads indicate the location of 5-HT-IR cells and the arrow shows the location of VAChT-IR cells. (B–D) Higher magnification of the region outlined in A (arrowhead: location of some 5-HT-IR cells). (E,F) Immunolabelling for VAChT (green) and a nuclear stain (DAPI, blue). (G,H) Immunolabelling for 5-HT (red) and a nuclear stain (DAPI, blue). The images from the red and green channels are shown separately and as a merged image. Scale bars: 250 µm (A), 25 µm (B–H).

We found cells containing VAChT in the pulmonary artery where the artery begins to branch (Fig. 8A arrow, E). These cells were oval (10.7±0.2 µm) with large nuclei and were arranged in large clusters close to the lumen of the vessel (Fig. 8E,F). Numerous serotonergic cells occurred along the first few centimetres of the artery where it emerges from the heart embedded in the vessel wall (Fig. 8A,B, arrowheads). 5-HT-IR cells were oval and 11.7±0.3 µm long. They were mostly arranged singly, but occasionally were found in groups of 2 or 3 (Fig. 8G,H). As in the other chemosensory areas described above, 5-HT and VAChT did not colocalize.

## DISCUSSION

This study identifies three peripheral chemoreceptive sites in the rattlesnake and defines the regulatory roles (cardiovascular or respiratory) of these different chemosensory sites. It also identifies putative oxygen-sensing cells in these regions based on the presence of neurotransmitters involved in oxygen chemotransduction in other vertebrates. The three O_2_-chemosensory sites are the area surrounding the carotid bifurcation and the bases of the aortic arch and pulmonary artery. In response to NaCN (mimicking low blood O_2_ levels), rattlesnakes increased ventilation and reduced the R–L intra-cardiac shunt (i.e. they decreased the fraction of blood that bypasses the lung). Each chemoreceptor site was effective in regulating shunt and ventilation. Putative oxygen-sensing cells containing serotonin were present in all chemosensory areas, but those containing acetylcholine were only found in the aorta and pulmonary artery. The results of our study allow us to compare the anatomy and function of chemosensory sites of rattlesnakes with those of other vertebrates.

### Limitations of the study

The complexity and invasiveness of the denervation surgeries precluded using the same animals for both intact and denervated experimental protocols. Only one animal in group 1 underwent both the intact and denervated experimental procedures.

We were also unable to separately denervate the aorta and pulmonary artery because the vagal innervation to these areas consists of numerous small branches that run closely together. We therefore cannot completely rule out the possibility that the reflex response produced by the pulmonary artery was not caused by recirculation of NaCN to the aorta. However, the similar response time and the different cardiovascular and ventilatory effects of pulmonary and aortic injections suggest that this was not the case. Furthermore, given the small concentration and volume of the NaCN injections, the bolus would have been diluted by the time it reached other chemosensory areas.

The ratio between pulmonary and systemic resistance is an important determinant of cardiac shunt (Overgaard et al., 2002). Unfortunately, consistent recording of pulmonary blood pressure proved challenging because of technical limitations. The small cannula (PE10) used was susceptible to clotting and difficult to maintain patent throughout the experiments. Therefore, mean pulmonary blood pressure, pulmonary pulse and pulmonary resistance were not calculated. All inferences about shunt control are deduced from the effects of NaCN on systemic resistance alone.

Ventilation was measured using subcutaneous impedance electrodes, with amplitude changes correlating to breath volume. This method accurately inferred breathing amplitude, as confirmed by direct volume assessment using masks on some snakes. Data were analysed as the percentage change from pre-NaCN injection values.

Tissue samples for immunohistochemical and histological analysis were fixed in PFA for varying durations, up to 7 days, depending on the collection date. To mitigate potential over-fixing, we collected tissue samples later in the study and stored them at 4°C until shipping. Although immunohistochemical results were positive, the extended fixation time may have slightly compromised antigenicity and image sharpness.

### Comparison with other studies

The values we obtained for cardiovascular variables under control conditions in our study are comparable with those obtained in other studies on unanaesthetized and resting rattlesnakes at room temperature (24–28°C) (Wang et al., 2001a; Skals et al., 2005; Taylor et al., 2009).

The presence of a net R–L or L–R shunt in resting snakes under control conditions was variable. A characteristic net R–L shunt as seen in resting turtles and toads (Shelton and Burggren, 1976; Overgaard et al., 2002; Krosniunas and Hicks, 2003; Andersen et al., 2003) was observed in some rattlesnakes, while in others a L–R shunt prevailed as reported in unanaesthetized resting rattlesnakes (Taylor et al., 2009). The net shunt, regardless of direction, was small. Nevertheless, the direction and magnitude of the cardiac shunt prior to the stimulus could potentially affect the response to NaCN injection. In the presence of central vascular shunts, arterial systemic blood is composed of systemic venous blood (deoxygenated blood) and blood returning from the lungs (oxygenated blood) (Wang et al., 1997). Thus, a R–L shunt will reduce the levels of oxygen in the arterial systemic blood (Wang and Hicks, 1996), which will presumably stimulate peripheral chemoreceptors and potentially affect their sensitivity to a new stimulus, such as NaCN. This has been reported in turtles, where NaCN injections in the pulmonary artery increased ventilation, but after exposure to hyperoxia for 1 h, NaCN did not have any effect on ventilation (Benchetrit et al., 1977).

### Location of peripheral chemoreceptor in rattlesnakes

We identified three chemosensory areas in rattlesnakes. We confirmed the chemosensory role of these areas by the absence of a reflex after selectively denervating and stimulating each site. Sham injections of saline (same volume and time of injection) further confirmed that responses were not due to stimulation of mechanoreceptors. We also verified that the vagus trunk remained intact after denervation of the different sites by stimulating a non-denervated area and confirming that changes in heart rate and blood flow still occurred.

Peripheral chemoreceptive areas in rattlesnakes were located at the carotid bifurcation, and at the bases of the aorta and pulmonary artery, derivatives of the 3rd, 4th and 6th pharyngeal arches, and were innervated by the glossopharyngeal/vagus trunk. Peripheral chemoreceptors are found in these same areas in turtles, frogs and toads, also innervated by the IX and/or X cranial nerves (Ishii et al., 1966, 1985a,b; Ishii and Ishii, 1986; Benchetrit et al., 1977; Lillo, 1980; Hoffman and de Souza, 1982; Reyes et al., 2014, 2015). Putative arterial oxygen-sensing cells identified in this study also share similar morphological characteristics and innervation with those found in other vertebrates, ranging from the neuroepithelial bodies of fish to the glomus cells in the carotid body of mammals. For instance, oxygen-sensing cells in rattlesnakes, as in other vertebrates, are oval with a diameter of 10–11 µm, and show large nuclei and the presence of vesicles with neurotransmitters (Ishii and Oosaki, 1969; Ishii et al., 1985b; Gonzalez et al., 1994; Jonz and Nurse, 2003, 2009, 2012; Campanucci and Nurse, 2007; Coolidge et al., 2008; Shakarchi et al., 2013; Reyes et al., 2014, 2015). As in red-eared sliders, chemosensory areas in rattlesnakes contain two different populations of putative oxygen-sensing cells, with distinct neurochemical content, arrangement and distribution (Reyes et al., 2015). One population consisted of 5-HT-IR cells arranged singly or in groups of 2–3 and mainly distributed in spongy tissue near lumen sinusoids in all three chemosensory areas. The second population comprised VAChT-IR cells arranged in large clusters and in proximity to the lumen of the aorta and pulmonary artery. Catecholamines were not found in any of the chemosensory areas. These striking similarities between the chemosensory areas in snakes and turtles support the notion that putative oxygen-sensing cells are highly conserved in reptiles.

In all lower tetrapods studied to date (amphibians and reptiles), all the extant derivatives of the pharyngeal arches contain chemosensitive sites. This also appears to be the case in rattlesnakes. It has been proposed that multiple O_2_-sensing chemoreceptors in distinct anatomical locations may be advantageous to animals with the ability to shunt, as distinct chemoreceptor groups will be exposed to blood with different composition (Wang et al., 1997; Milsom and Burleson, 2007). For instance, carotid and aortic chemoreceptors will be exposed to arterial blood with oxygen levels that will depend on the degree of admixture, while chemoreceptors in the pulmonary artery will be exposed to mixed venous blood and venous oxygen levels will be determined by systemic oxygen delivery (blood flow and O_2_ content of arterial blood) relative to tissue oxygen uptake (Wang et al., 1997).

### Regulatory roles and stimulus specificity of chemoreceptor groups

#### Effects of chemoreceptor stimulation on respiratory control

NaCN injection at each of the three chemosensitive sites in the rattlesnakes increased ventilation. Changes in both *f*_R_ and *V*_amp_ were responsible for the increase in ventilation associated with injections into the carotid and aortic chemosensitive areas, while the effect of injections into the pulmonary chemoreceptor area was produced primarily by changes in *V*_amp_ alone. The peak *f*_R_ response occurred earlier than the *V*_amp_ response following stimulation at all sites. The magnitude of the ventilatory response and the respiratory pattern (tidal volume or breathing frequency) used to produce this response under conditions of low oxygen are highly variable among different species of reptiles (Shelton et al., 1986). For instance, garter snakes exposed to hypoxia increase ventilation mainly by increasing tidal volume accompanied by a small rise in breathing frequency (Bartlett and Birchard, 1983). In contrast, the hypoxic ventilatory response of the diamondback water snake consists of an increase in breathing frequency and a fall in tidal volume, such that the increase in ventilation is small (Gratz, 1979). Our findings indicate that the hypoxic ventilatory response in the rattlesnake consists of an amplitude and frequency response and that the net effect arises from a balance of changes to the breathing pattern produced by each chemoreceptor group.

The ventilatory response of pulmonary and carotid chemoreceptor stimulation was faster and slightly larger than that of the aortic chemoreceptors. The pulmonary artery of turtles has also been found to be an important chemosensory site, as NaCN injection in this area had a major effect on ventilation compared with the effects of stimulating the aorta and truncus arteriosus (Benchetrit et al., 1977). The time course of the contributions by each site also varied, with that arising from stimulation of the carotid chemosensitive area being more transient.

#### Effects of chemoreceptor stimulation on cardiovascular control

NaCN injection at all chemosensory sites caused a fall in *Q̇*_sys_, and increases in *f*_H_, *R*_sys_ and *Q̇*_pul_/*Q̇*_sys_, so that the R–L shunt was reduced. There were, however, some differences in the reflexes produced by the distinct chemoreceptive sites. Stimulation of the carotid and aortic chemosensory areas produced a rise in *Q̇*_pul_, but no change in blood pressure or cardiac output. The cardiovascular effects of NaCN injection at pulmonary chemoreceptive sites were large and consisted of a fall in *Q̇*_pul_, cardiac output and blood pressure.

A reduction of the R–L shunt during hypoxia has been well documented in reptiles (Wang and Hicks, 1996; Andersen et al., 2003); therefore, it is not surprising that NaCN stimulation of all chemosensory sites in rattlesnakes caused a reduction in shunt. Changes in shunt pattern are effective in adjusting arterial blood gases in reptiles and amphibians (Burggren and Shelton, 1979; Wood, 1982, 1984; Burggren, 1988; Burggren et al., 1989; Wang and Hicks, 1996; Wang et al., 1997).

##### Mechanisms of cardiac shunt regulation

The reductions in R–L shunt after chemoreceptor stimulation in our study were consistently achieved by a drop in systemic blood flow accomplished through an increase in systemic resistance. The increase in *R*_sys_ and concomitant reduction in the *Q̇*_sys_ has been attributed to increased sympathetic tone in anaesthetized rattlesnakes and turtles (Overgaard et al., 2002; Galli et al., 2007). In unanaesthetized and decerebrated rattlesnakes, adrenergic stimulation causes varied vascular changes, reducing systemic blood flow and compliance while increasing resistance and blood pressure. Changes in pulmonary arterial compliance and resistance can balance the R–L shunt (Castro et al., 2023). Thus, the increased pulmonary blood flow after carotid and aortic chemoreceptor stimulation likely resulted from decreased pulmonary resistance. Unfortunately, we were unable to measure *R*_pul_ in this study, but it is well known that regulation of cardiac shunts in reptiles is mainly achieved by cholinergic vagal control of the smooth muscle surrounding the pulmonary artery (Burggren, 1977; Milsom et al., 1977; Hicks and Comeau, 1994; Hicks, 1998; Wang et al., 2001b). Adrenergic stimulation of the systemic circulation and release of vagal tone on the pulmonary circulation together will decrease *Q̇*_sys_ and increase *Q̇*_pul_, respectively, reducing the R–L shunt (Hicks, 1994; Comeau and Hicks, 1994; Overgaard et al., 2002). The reduction in the R–L shunt in our study was achieved by a redistribution of blood flow such that cardiac output did not change. Under these conditions, the oxygen content of systemic arterial blood should have increased while systemic flow decreased.

Activation of pulmonary chemoreceptors, however, significantly reduced both blood flow and cardiac output. As the fall in *Q̇*_sys_ was larger than the fall in *Q̇*_pul_, the net effect was still a reduction in the R–L shunt. Pulmonary chemoreceptors regulated arterial oxygen by adjustments in total blood flow and the degree of shunt.

We do not know what the net effect would be of stimulation at all three sites simultaneously in the rattlesnakes, but our findings indicate that regulation of *Q̇*_sys_ plays a key role in the control of net cardiac shunt in rattlesnakes.

##### Response time of chemoreceptor stimulation

Changes of cardiovascular and respiratory variables after chemoreceptor stimulation generally started after a short delay (30 and 50–120 s). However, maximum (significant) effects were not reached until later and this varied between physiological variables and between chemoreceptive sites. The time needed to reach a maximum change in ventilatory and cardiovascular variables in our study was longer than the recirculation time, but denervation of specific chemosensory areas allowed us to exclude the possibility of stimulation of other areas by recirculation of NaCN.

The effects of NaCN infusion into the chemosensory areas of rattlesnakes were also delayed compared with those of NaCN injection into the carotid labyrinth and pulmocutaneous artery of frogs and toads (Lillo, 1980; Wang et al., 2004). Species differences, the concentration of NaCN used or the fact that the earlier experiments were performed on lightly anaesthetized frogs and decerebrated and unidirectionally ventilated toads could explain the differences in time response between studies. However, in anaesthetized turtles, maximum increase in nerve discharge occurred after 1–1.5 min post-NaCN injection (Ishii et al., 1985b). The time response in anaesthetized turtles is significantly longer than the emergence of a reflex response in anaesthetized and decerebrated amphibians, suggesting that in reptiles, both signal chemotransduction (turtles) and reflex production (rattlesnakes) take longer than in other vertebrates.

##### Reflex roles of peripheral chemoreceptors

One of the objectives of this study was to determine whether distinct chemoreceptor groups specifically controlled the cardiovascular versus respiratory systems in the rattlesnake. We hypothesized that aortic and pulmonary chemoreceptors would primarily regulate the degree of intra-cardiac shunt, while carotid chemoreceptors would primarily regulate ventilation. Here, we demonstrate that all three anatomically separate peripheral chemoreceptive sites in rattlesnakes have similar reflex roles in cardiovascular and respiratory control.

Previous studies on toads and turtles showed that reductions in arterial oxygen-carrying capacity produced an increase in heart rate and pulmonary blood flow while ventilation remained unchanged, and a decrease in arterial oxygen partial pressure increased ventilation (Wang et al., 1994, 1997; Branco and Glass, 1995; Andersen et al., 2003). Several studies found correlations between the hypoxic ventilatory response in reptiles and changes in oxygen content rather than *P*_O_2__ (Glass et al., 1983). To explain these observations, Wang and collaborators (1997) postulated two hypotheses: (1) the existence of an O_2_ content-sensitive chemoreceptor or (2) the presence of a chemoreceptor group in the pulmocutaneous and pulmonary arteries of amphibians and reptiles that specifically affects the cardiovascular system (Wang et al., 1997). The aortic bodies of mammals have been shown to be sensitive to changes in oxygen content and to mainly regulate the cardiovascular system (Jones and de Burgh Daly, 1997), while the carotid bodies are *P*_O_2__ sensitive and are predominantly responsible for ventilatory control (Lahiri et al., 1981). Aortic bodies are under-perfused relative to their oxygen consumption, which makes them sensitive to changes in oxygen concentration as well as *P*_O_2__. The presence of chemoreceptors in the pulmocutaneous or pulmonary arteries of amphibians, turtles and snakes has been demonstrated (Lillo, 1980; Hoffman and de Souza, 1982; Ishii et al., 1985a,b; Wang et al., 2004; Reyes et al., 2014, 2015; present study). Based on these observations, both hypotheses are attractive. That both ventilatory and cardiovascular adjustments occurred after stimulation of each chemosensory sites in rattlesnakes (present study) and toads (Wang et al., 2004) suggests that chemoreceptor groups with distinct reflex roles do not exist in reptiles and amphibians.

Furthermore, our data do not support the presence of a group of receptors within the aorta or pulmonary artery that are under-perfused relative to their oxygen consumption, making them sensitive to changes in oxygen concentration as wells as *P*_O_2__. We found that all populations of putative oxygen-sensing cells in each of the chemosensory areas of snakes have similar distributions and are all close to luminal blood flow (see ‘Distribution and neurochemical content of putative oxygen-sensing cells’, below). The same is true for turtles (Reyes et al., 2015) and for the aorta and carotid labyrinth of frogs (Reyes et al., 2014).

That said, we still cannot rule out the possibility of some chemoreceptors at one of these sites having different stimulus modalities as stimulation with NaCN does not allow for differentiation between reductions in oxygen content and oxygen tension (*P*_O_2__). All we can conclude is that some receptors at all chemoreceptive sites respond to NaCN by eliciting cardiovascular and ventilatory adjustments.

### Distribution and neurochemical content of putative oxygen-sensing cells

We found the presence of vesicular acetylcholine transporter and serotonin, but not catecholamines in putative oxygen-sensing cells in the rattlesnake. VAChT-IR cells were grouped together in spongy tissue towards the lumen of the aorta and pulmonary artery. These cells were more often found in the distal segments of the chemosensitive areas of this blood vessel. Serotonergic cells arranged singly or in small clusters of 2–3 were found throughout the chemosensitive areas of the carotid bifurcation, aorta and pulmonary artery. Their density, however, seemed to be higher in spongy-like tissue towards the heart end of the chemosensitive areas of the aorta and pulmonary artery. Although these neurotransmitters never colocalized, the two cell types occasionally overlapped in the chemosensitive areas of the blood vessels. Putative oxygen-sensing cells containing either serotonin or acetylcholine were also present in all the chemosensory areas of red-eared sliders (Reyes et al., 2015). The morphology, size and arrangement of putative serotonergic and cholinergic O_2_-sensing cells in turtles and snakes are remarkably similar and coincide with anatomical features reported for chemoreceptor cells in other vertebrates (Ishii and Oosaki, 1969; Ishii et al., 1985b; Gonzalez et al., 1994; Jonz and Nurse, 2003; Campanucci and Nurse, 2007; Coolidge et al., 2008; Shakarchi et al., 2013).

There are, however, some differences in the distribution and neurochemical content of chemosensory areas between turtles and snakes. First, in rattlesnakes, there was no evidence of the polygonal VAChT-IR cells present at the outflow of the aorta and pulmonary arteries in turtles. It has been proposed that these cells are involved in vasomotor regulation (Reyes et al., 2015). Rattlesnakes have large R–L shunts (Wang et al., 1998; Galli et al., 2005; Taylor et al., 2009) and, in reptiles, changes in shunt pattern are regulated by cholinergic mediated constriction of the pulmonary artery (Burggren, 1977, 1987; Milsom et al., 1977; Hicks, 1994, 1998; Wang et al., 2001b); therefore, we would have expected similar Ach-positive cells at the outflow of the aorta and pulmonary artery. As mentioned above, clusters of oval VAChT-IR cells are present in the pulmonary artery and aorta of snakes, but their location is by the lumen, not in close contact with smooth muscle, and distal to the heart. These observations taken together with their resemblance to putative O_2_-sensing cells in turtles (Reyes et al., 2015) suggest a chemosensory role. We cannot rule out, however, that VAChT-IR cells in snakes have a dual function in afferent and efferent control. Second, we found no sign of VAChT-IR cells in the carotid bifurcation. Ovoid 5-HT-IR cells in the pulmonary vasculature of the snake *Acrochordus granulates* have been postulated to be chemosensory as well (Donald and Lillywhite, 1989). Their morphology, size (10–16 µm in diameter) and arrangement correspond to serotonergic cells in the carotid bifurcation, aorta and pulmonary artery of rattlesnakes in our study.

Given the anatomical similarities of VAChT and 5-HT-IR cells in rattlesnakes to chemoreceptor cells in other vertebrates and that serotonin and acetylcholine have been proposed to be involved in O_2_ sensing in mammals and fish (Dunel-Erb et al., 1982; Gonzalez, et al., 1994; Zaccone et al., 1997; Jonz and Nurse, 2003; Nurse, 2005, 2010; Porteus et al., 2012; Shakarchi et al., 2013; Zachar and Jonz, 2012), we postulate that both serotonergic and cholinergic cell types in rattlesnakes are O_2_ sensors. All data collected to date indicate that putative oxygen-sensing cells in reptiles share the same neurochemical content. Serotonin is present in peripheral chemoreceptors of all vertebrates studied and the presence of catecholamines and acetylcholine varies between groups. Our data on rattlesnakes support the observation of a trend to increase the number of neurotransmitters involved in oxygen signal transduction from fish to amphibians and reptiles to mammals (Milsom and Burleson, 2007).

### Conclusion

This study examined O_2_ chemoreceptors in rattlesnakes, detailing their location, cardiorespiratory roles and putative neurochemical profiles. Their location in derivatives of pharyngeal arches 3, 4 and 6 (carotid bifurcation, aorta and pulmonary artery, respectively) is the same as in turtles and amphibians. Having multiple chemoreceptor groups that sense arterial (carotid and aortic chemoreceptors) or mixed venous blood (pulmonary chemoreceptors) may construe an advantage to animals with intra-cardiac shunts. We determined the regulatory roles (cardiovascular or respiratory) of the different chemoreceptive areas and identified putative oxygen-sensing cells in these regions. NaCN stimulation of all sites, mimicking anoxia, triggered adjustments in cardiac shunt and ventilation. That all chemosensory sites produced changes in cardiac shunt fraction suggests that adjustments of the cardiovascular system through changes in blood flow are important in regulating arterial blood gases. Rattlesnakes, like most air breathers, rarely encounter hypoxia, but given their undivided ventricle, hypoxaemia is not uncommon. Consequently, shunt regulation is more critical than ventilation changes. Regardless, in the presence of low blood O_2_ levels, rattlesnakes increase ventilation, reduce the R–L shunt (decreasing the fraction of blood bypassing the lung) and increase heart rate to maintain blood gas homeostasis. Furthermore, acetylcholine and serotonin may be involved in oxygen chemotransduction, as we found populations of putative chemoreceptor cells containing these neurotransmitters. These cells resemble those in other vertebrates, particularly turtles, in arrangement and neurochemical content.

## Supplementary Material

10.1242/jexbio.249222_sup1 Supplementary information
