# Supplementary material for: Distribution and role of peripheral arterial chemoreceptors in cardio-respiratory control of the South American rattlesnake (Crotalus durissus)
Source: J Exp Biol. 2025 Feb 20;228(4):JEB249222. doi: 10.1242/jeb.249222 (PMC11883273; doi:10.1242/jeb.249222)
Supplement: Supplementary information [file jexbio-228-249222-s1.pdf]

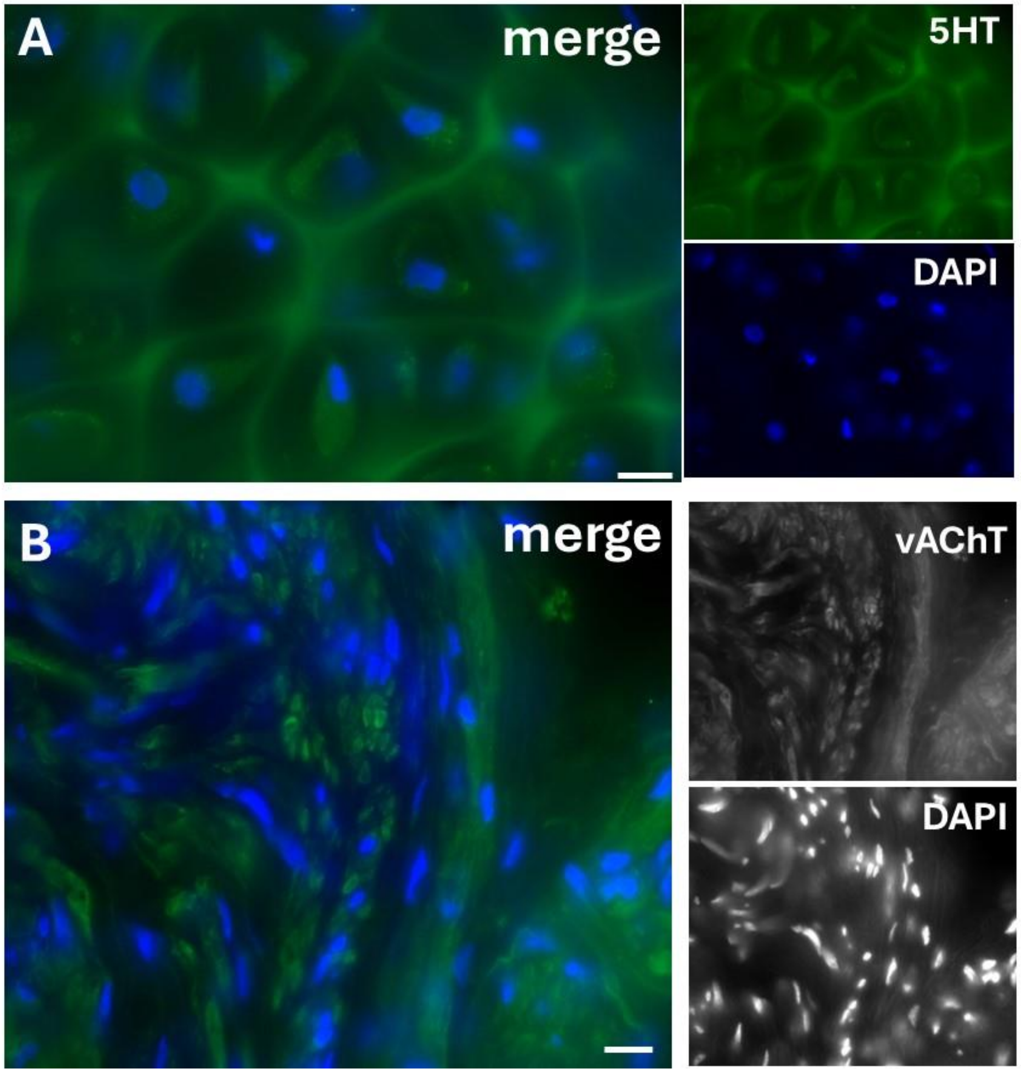

Fig. S1. Positive controls for serotonin (5-HT) and vesicular acetylcholine transporter (vAChT) in the lung and jugular ganglia of *Crotalus durissus*. A: Double immunolabeling for 5-HT (green) and cell nuclei (DAPI, blue), showing serotonergic cells in the lung. B: Double immunolabeling for vAChT (green) and cell nuclei (DAPI, blue) in the jugular ganglia. The image from green and blue channels are sown separately and as a merged.

Table S1. Effects of saline injections (2ml) on pulmonary blood flow ($\dot{Q}_{pul}$), systemic blood flow ($\dot{Q}_{sys}$), heart rate (f_H), mean arterial pressure (MAP), total ventilation ($\dot{V}_{Tot}$), breathing frequency (f_R) and amplitude (V_{AMP}). Values: mean ± s.e.m. (N=9, carotid group; N=8, aortic group and N=9, pulmonary group). $\dot{V}_{Tot}$ and V_{AMP} are expressed as percentage values relative to the pre-injection (control) values. Saline injections had no significant effect relative to pre-injection value (control, Holm-Sidak pairwise comparison). Ca: carotid artery, Ao: aorta, PA: pulmonary artery.

Time	$\dot{Q}_{pul}$ (ml min^{-1} kg^{-1})			$\dot{Q}_{sys}$ (ml min^{-1} kg^{-1})			f_H (min^{-1})			MAP (mmHg)		
**Saline (2ml)**	**Ca**	**Ao**	**PA**	**Ca**	**Ao**	**PA**	**Ca**	**Ao**	**PA**	**Ca**	**Ao**	**PA**
control	27.3±3.4	25.7±4.9	24.8±2.8	28.1±5.5	38.0±10.8	39.7±13.8	37.1±2.9	38.6±3.4	37.3±5.0	41.9±5.1	35.0±3.3	33.3±3.4
60s	25.4±3.7	25.7±5.0	24.2±3.0	28.6±5.7	37.9±10.2	41.7±13.1	37.3±3.0	37.6±3.4	37.8±4.7	42.8±5.3	35.3±3.4	33.4±3.1
120s	26.7±3.6	25.4±4.5	23.6±3.2	27.8±5.9	35.2±7.8	38.0±13.5	37.6±2.9	38.1±3.8	37.0±4.7	43.7±5.4	34.6±3.3	32.3±2.9
180s	25.5±3.5	25.4±4.3	25.1±3.3	28.3±6.1	32.9±6.4	39.6±13.4	37.6±2.8	38.0±3.3	37.9±4.4	44.3±5.4	35.2±3.3	32.3±3.1
240s	24.7±3.2	23.9±4.5	24.6±3.2	27.9±5.9	32.6±6.5	40.1±13.4	37.4±2.8	38.1±3.5	38.2±4.6	44.5±5.2	36.6±3.7	32.8±3.2
300s	24.6±3.3	25.7±4.0	25.3±3.2	27.6±5.6	32.8±6.9	40.3±13.5	37.4±3.0	38.6±3.4	38.6±4.7	44.8±5.3	35.8±3.6	33.4±3.5

Time	V_{Tot} (% change)			f_R (min^{-1})			V_{AMP} (% change)		
**Saline (2ml)**	**Ca**	**Ao**	**PA**	**Ca**	**Ao**	**PA**	**Ca**	**Ao**	**PA**
control	100	100	100	3.4±0.5	4.4±0.7	3.6±0.7	100	100	100
60s	135.5±26.8	151.4±49.9	146.1±19.7	3.3±0.6	4.9±0.9	3.6±0.7	133.4±21.5	109.9±22.0	147.5±20.9
120s	146.6±36.2	65.9±18.5	104.4±19.1	3.2±0.6	3.4±0.9	3.2±0.7	181.1±42.9	81.9±19.2	111.5±13.6
180s	166.3±31.9	52.7±21.3	111.3±22.6	3.6±0.6	3.2±1.3	3.3±0.7	171.9±36.8	69.7±25.0	107.6±14.4
240s	128.6±24.0	76.4±20.7	112.4±18.1	2.9±0.6	2.6±0.7	3.6±0.8	187.8±40.4	96.3±27.6	99.1±10.9
300s	165.6±37.9	68.4±13.2	72.4±15.5	3.0±0.6	3.0±1.0	2.3±0.5	201.7±38.4	95.1±30.3	95.9±14.4

Journal of Experimental Biology • Supplementary information
